# The Utility of Blood Culture Fluid for the Molecular Diagnosis of *Leptospira*: A Prospective Evaluation

**DOI:** 10.4269/ajtmh.15-0674

**Published:** 2016-04-06

**Authors:** Sabine Dittrich, William E. Rudgard, Kate L. Woods, Joy Silisouk, Weerawat Phuklia, Viengmon Davong, Manivanh Vongsouvath, Koukeo Phommasone, Sayaphet Rattanavong, Michael Knappik, Scott B. Craig, Steven L. Weier, Suhella M. Tulsiani, David A. B. Dance, Paul N. Newton

**Affiliations:** Lao-Oxford-Mahosot Hospital-Wellcome Trust Research Unit, Microbiology Laboratory, Mahosot Hospital, Vientiane, Lao PDR; Centre for Tropical Medicine and Global Health, Nuffield Department of Medicine Research Building, Nuffield Department of Medicine, University of Oxford, Oxford, United Kingdom; Public Health England, Colindale, London, United Kingdom; Faculty of Science Health, Education and Engineering, University of the Sunshine Coast, Sippy Downs, Queensland, Australia; Queensland Health Forensic and Scientific Services, WHO Collaborating Centre for Reference and Research on Leptospirosis, Brisbane, Queensland, Australia; Faculty of Health, Queensland University of Technology, Brisbane, Queensland, Australia; Copenhagen Centre for Disaster Research, Global Health Section, Department of Public Health, University of Copenhagen, Copenhagen, Denmark

## Abstract

Leptospirosis is an important zoonosis worldwide, with infections occurring after exposure to contaminated water. Despite being a global problem, laboratory diagnosis remains difficult with culture results taking up to 3 months, serology being retrospective by nature, and polymerase chain reaction showing limited sensitivity. Leptospira have been shown to survive and multiply in blood culture media, and we hypothesized that extracting DNA from incubated blood culture fluid (BCF), followed by quantitative real-time polymerase chain reaction (qPCR) could improve the accuracy and speed of leptospira diagnosis. We assessed this retrospectively, using preincubated BCF of *Leptospira* spp. positive (*N* = 109) and negative (*N* = 63) febrile patients in Vientiane, Lao PDR. The final method showed promising sensitivities of 66% (95% confidence interval [CI]: 55–76) and 59% (95% CI: 49–68) compared with direct or direct and indirect testing combined, as the respective reference standards (specificities > 95%). Despite these promising diagnostic parameters, a subsequent prospective evaluation in a Lao hospital population (*N* = 352) showed that the sensitivity was very low (∼30%) compared with qPCR on venous blood samples. The disappointingly low sensitivity does suggest that venous blood samples are preferable for the clinical microbiology laboratory, although BCF might be an alternative if leptospirosis is only suspected postadmission after antibiotics have been used.

## Introduction

Leptospirosis is a worldwide zoonotic disease, particularly common in rural tropical regions associated with contaminated water and soil exposure during heavy rainfall and occupational risks.^1^ Poor living conditions in urban slums also contribute to an apparently increasing numbers of cases.^2^ Leptospiral infections are difficult to differentiate clinically from many other important causes of fever in the tropics, for example, rickettsial infections, typhoid, dengue, and malaria. In the United Kingdom and other temperate zones, cases may be indigenously associated with occupational or recreational risk factors or acquired overseas, predominantly in Southeast Asia or Central America.^3^ Leptospirosis usually presents as a nonspecific febrile illness, but may progress to serious renal, pulmonary, and central nervous system complications, with a case fatality rate of up to 40%.^4^,^5^ A lack of accurate diagnostic tools makes individual patient diagnosis to guide treatment very difficult.^6^–^9^ Serological methods are of limited use at admission, particularly in populations with regular exposure to the pathogen in whom only a 4-fold rise between admission and convalescent samples indicates recent infection. The organism is slow-growing and fastidious, requiring specialized prolonged culture methods and media^10^; hence for timely diagnosis, molecular tools have emerged as the methodologies of choice.^11^ Several molecular methods have been developed mostly using venous (ethylenediaminetetraacetic acid [EDTA]) blood.^9^,^11^,^12^ We recently demonstrated the utility of molecular diagnosis of *Orientia tsutsugamushi*, the agent of scrub typhus, from blood culture fluid (BCF) after 24-hour incubation (S. Dittrich, submitted). It has also been shown that *Leptospira* spp. survive in conventional blood culture media for up to 14 days but were not detected by conventional alert systems.^13^,^14^ Villumsen and others^14^ explored the use of BCF in place of venous blood as a sample for early leptospira diagnosis even days after antibiotic therapy initiation. Such techniques, especially if there was bacterial multiplication in blood cultures before quantitative real-time polymerase chain reaction (qPCR), could increase the sensitivity of assays and give additional sample matrices to work with. We, therefore, performed an initial pilot proof-of-principle and then a prospective study to determine whether blood culture bottles incubated for 24 hours in the Microbiology Laboratory of Mahosot Hospital (Vientiane, Lao PDR) could be used for the early diagnosis of leptospira by qPCR.

## Materials and Methods

### Study population.

The methodology was optimized using *Leptospira* spp. qPCR positive^9^ and negative samples, collected as part of studies in different geographical regions of the Lao PDR (Laos): Vientiane (*N* = 65), Luang Namtha (*N* = 51), and Salavan (*N* = 18). Samples had tested positive by qPCR (*N* = 18), culture (*N* = 73), or microscopic agglutination tests (MATs; *N* = 18) as part of published investigations.^5^,^15^

The subsequent prospective study was conducted between May and August 2014 using consecutive patient samples (*N* = 363), submitted to the Microbiology Laboratory of Mahosot Hospital (102.6119°E, 17.9604°N). The inclusion criteria were fever and/or headache, rash, eschar, myalgia, arthralgia, meningitis, encephalitis, respiratory symptoms, jaundice, and acute renal failure. Samples were collected on the day of admission for molecular diagnostics (serum, EDTA buffy coat, EDTA whole blood), leptospiral culture (blood clot), serology (serum), and two aerobic blood culture bottles. The aerobic blood culture bottles (Pharmaceutical Factory No. 2, Vientiane, Laos), which contain tryptic hydrolysate of casein and soy peptone with sodium polyanethol sulfonate (SPS), were inoculated with blood (> 15 years: 5 mL; 1–15 years: 2 mL; < 1 year: 1 mL), vented, and incubated at 37°C for 7 days.^16^ Bottles were manually checked for turbidity and blind subcultures (blood, chocolate, and MacConkey agars) performed after 24 hours and 7 days of incubation.^16^

BCF was collected from all blood cultures after 24-hour incubation. Bottles were repeatedly inverted until blood and media were homogeneously mixed, and 0.5 mL BCF from each blood culture bottle was transferred into another tube (total volume 1–1.5 mL of BCF per patient). Samples were stored at 4°C for up to 7 days and at −20°C for long term.

All study patients provided written informed consent prior to sample collection, with parents or guardians of any child participant providing informed consent on their behalf. Ethical approval for all investigations was granted by Oxford Tropical Research Ethics Committee (University of Oxford, United Kingdom) and the Faculty of Medical Sciences Committee (University of Health Sciences, Lao PDR).

### Sample processing.

EDTA blood samples (buffy coat, whole blood) and serum were processed using the Qiagen Blood Mini Kit (Qiagen, Germany) as described.^15^ BCF (0.5 mL) were spun for 10 minutes at 15,900× *g* to pellet free bacteria and cells, but ∼200 μL of the supernatant were discarded before proceeding as described.^14^ BCF was processed using the benzyl alcohol–based method, to remove, combined with the guanidine hydrochloride lysis/Qiagen Blood Mini Kit extraction, and obtained a final elution of 50 μL.^14^

### Molecular detection.

*Leptospira* spp. detection was performed using a published qPCR targeting the *rrs* gene (16S rRNA).^9^ In each run, 5 μL of template DNA (BCF, buffy coat, whole blood or serum) were added, and when using BCF-DNA, 40 mg/mL BSA was included to overcome residual inhibition due to SPS.^17^ Each run contained a standard curve of genomic *Leptospira* DNA (1 genome equivalent (GE)/μL to 10^3^ GE/μL; clinical isolate from Laos; assumed genome size, 4.7 Mb). Nontemplate controls were added to each run and were always negative; that is, no amplification was detected. All runs were performed using a Rotorgene 6000 (Qiagen).

### Analysis.

Threshold analysis was performed using RotorGene^™^ 6000 software (Version 1.7; Qiagen, Hilden, Germany). Receiver operator characteristics (ROC) analysis and figures were prepared using GraphPad Prism (Version 6.0c; San Diego, CA). In accordance with published guidelines, qPCR results were only considered positive with a Cq-value of ≤ 40 unless otherwise stated.^18^ MATs were performed by the WHO/FAO/OIE (World Health Organization/Food and Agriculture Organization of the United Nations/World Organization for Animal Health) Collaborating Center for Reference and Research on Leptospirosis, Australia.^15^ A 4-fold increase in titer between admission and convalescent samples was considered as an evidence of acute infection, whereas a 2-fold increase/decrease and a titer ≥ 1:400 were considered as “evidence of probable or recent infection.” Diagnostic accuracy was calculated in comparison to culture results, culture + qPCR, and with culture + qPCR + 4-fold rise in MAT titer.

## Results

### Pilot study.

**Figure 1.**
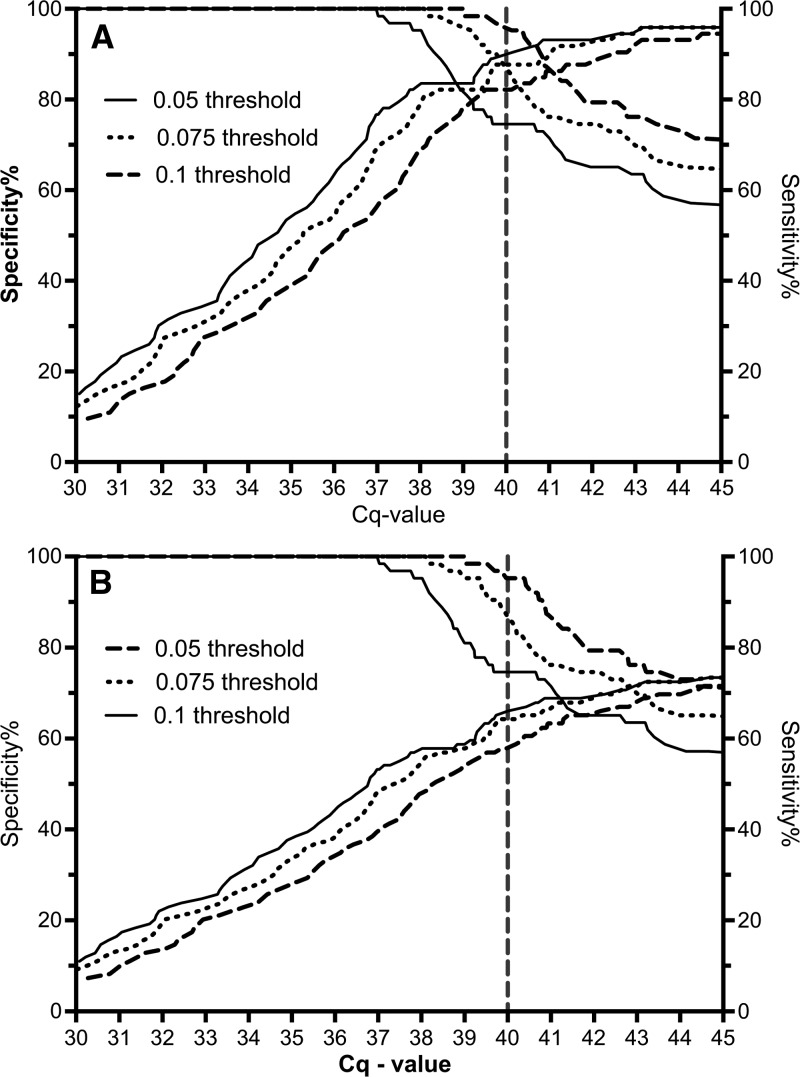
Sensitivity and specificity characteristics plotted for different threshold values. Real-time quantitative polymerase chain reaction (qPCR) assays were analyzed using three different fluorescent thresholds (0.05, 0.075, 0.1), which were set manually. Receiver operator characteristics (ROC) analysis was used to determine sensitivity and specificity values at different Cq-values. Using (**A**) culture and (**B**) both culture and qPCR (blood) as gold standards for the ROC analysis.

The retrospective study aimed to pilot the use of 24-hour incubated BCF samples for molecular diagnosis and develop appropriate local qPCR threshold values. In total, BCFs from 109 *Leptospira* spp. qPCR, culture, or MAT positive and 63 *Leptospira* spp. qPCR and MAT negative patients were used. Each qPCR run was analyzed using three fluorescent threshold values (0.05, 0.075, 0.1), to include or exclude low positive samples and optimize the assay sensitivity and specificity accordingly (Figure 1

). Using ROC analysis, a threshold level of 0.1 gave the best balance between sensitivity and specificity with an estimated sensitivity of 65.9% (95% confidence interval [CI]: 55.3–75.6) and specificity of 95.2% (95% CI: 86.7–99.0) when compared with culture and qPCR as a combined gold standard (Table 1). Reducing the threshold and increasing the inclusion Cq would have led to a sensitivity of close to 100% (gold standard: culture) while the specificity would fall to ∼70% (Figure 1), that is unacceptable for both clinical diagnostics and public health investigations.

The defined analysis criteria (Cq ≤ 40; fluorescent threshold: 0.1) were subsequently used in a prospective study to investigate the real-life diagnostic characteristics in a cohort of Lao hospital patients.

### Prospective evaluation.

Over the study period, 363 consecutive patients meeting the inclusion criteria were recruited. The majority was male (217/363; 60%), and the median age was 40 years (range: 1–92) with a median of 6 days (0–90 days) of illness prior to admission. For 352 (97%) patients, BCF and venous blood sample were available and of those 10 (2.8%) patients were positive for *Leptospira* spp. by molecular assays on admission blood samples (buffy coat, serum, or whole blood). When BCF after 24-hour incubation was used as a template for the qPCR, 6/352 (1.7%) patients were positive by qPCR. Out of these six, only three (50%) patients' samples were also positive by qPCR on one or more blood compartments and/or urine (data not shown), whereas seven (70%) patients with positive blood samples (serum, buffy coat, whole blood) were negative by qPCR on BCF. The three patients who were only positive by qPCR on BCF were also positive for other organisms, either by PCR on EDTA blood (*Rickettsia* spp.)^20^ or blood culture (coagulase-negative *Staphylococcus* and *Burkholderia cepacia*)^16^ and their sera were negative by MAT for *Leptospira* spp. infections. The final sensitivity of qPCR on BCF when calculated in comparison to venous blood (buffy coat, serum, whole blood) was 30% with a specificity of 99%. The positive predictive value (PPV) was only 43%, whereas the negative predictive value was very high (98%) (Table 1).

In addition to using the predefined criteria (Cq ≤ 40, threshold: 0.1), including positives with higher Cq-values was also explored (Table 2). Although the sensitivity doubled to 50% when including all positives with Cq ≤ 45,^21^ the specificity dropped to 88%. In this prospective study this would have wrongly identified 41 patients as having leptospirosis, overestimating the leptospira frequency in the hospital population by approximately 4-fold (∼3% versus 13%). Only one of those 41 false-positive patients (Cq-cutoff: ≤ 45) had serological evidence for a recent *Leptospira* spp. infection by MAT.

## Discussion

The need for improved molecular tools for *Leptospira* spp. diagnosis is reflected in the multitude of new molecular tests that have been recently developed.^21^–^24^ BCF as a sample for direct detection of leptospirosis has been proposed,^14^ but no detailed data on its utility in a clinical laboratory are available. Our study aimed to establish the optimal methodology and subsequently to evaluate the method in febrile inpatients. The initial pilot study showed promising sensitivities of 82%, 90%, 66%, and 59% compared with culture, PCR, culture + PCR, or culture + PCR + MAT, as the respective reference standards. These diagnostic characteristics compare very well with the published sensitivity of the qPCR when used with whole blood (∼55%),^25^ suggesting that BCF might represent a valid and possibly superior blood sample compared with uncultured EDTA blood. However, in the subsequent prospective evaluation in a Lao hospital population, the BCF results underestimated leptospiral incidence in comparison to qPCR of uncultured EDTA blood. Seventy percent of *Leptospira* spp. positive patients would have been missed when using only BCF as a sample. Further to highlighting issues with the use of BCF, our results underline the complexity of leptospira diagnosis with none of the qPCR positive patients showing evidence for leptospira infection by the serological gold standard.^24^,^25^

The qPCR assay used in this study targets the 16S rRNA gene of *Leptospira* spp., and the ubiquity of this target can result in reduced specificity. A number of studies have described this issue^9^,^23^,^26^ and our results confirm those findings as all of the actual false-positive samples had other bacteria present in the blood or blood culture sample, even though two of these are likely to have been contaminants. The specificity and PPV dropped further when the Cq-cutoff was extended to 42 or 45, consistent with the Minimum Information for Publication of Quantitative Real-Time PCR Experiments (MIQE) guidelines that suggest that Cq-values > 40 are suspect and should not be considered positive.^18^ This trade-off with the sensitivity has to be accepted, as overestimating disease incidence and initiating the wrong treatment would have adverse consequences for patients.

Using incubated BCF samples seems advantageous for other pathogens (*O. tsutsugamushi*) (S. Dittrich, submitted), but this does not appear to be the case when using a 16S rRNA-based qPCR system for leptospirosis. Blood culture bottles containing glycerol or fatty acids were shown to be conducive to leptospiral growth^13^; hence, future investigations using supplemented blood culture media may be illuminating.

In conclusion, the study shows that, despite the initial promising results, BCF, when combined with a 16S rRNA-targeting assay, does not seem sufficiently sensitive and specific for routine molecular diagnosis of leptospirosis. The results underline the need for increased investment into improved diagnostic tools for this important global pathogen.

## Figures and Tables

**Table 1:** Diagnostic characteristics obtained by ROC analysis for three different fluorescent cutoffs and different gold standard comparators

	Sensitivity (95% CI)	Specificity (95% CI)	Positive/negative*	AUC† (95% CI)
Threshold: 0.05				
Culture	89.0% (79.5–95.2)	74.6% (62.1–84.7)	73/63	0.94
qPCR	84.2% (60.4–96.6)	25.4% (15.3–37.9)	19/63	0.57
Culture + qPCR	74.7% (64.5–83.3)	74.6% (62.1–84.7)	91/63	0.82
Culture + qPCR + MAT	66.1% (56.4–74.9)	74.6% (62.1–84.7)	109/63	0.76
Threshold: 0.075				
Culture	87.7% (77.9–94.2)	87.3% (76.5–94.3)	73/63	0.95
qPCR	84.2% (60.4–96.6)	25.4% (15.3–37.9)	19/63	0.57
Culture + qPCR	72.5% (62.2–81.4)	87.3% (76.5–94.3)	91/63	0.85
Culture + qPCR + MAT	64.2% (54.5–73.2)	87.3% (76.5–94.3)	109/63	0.79
Threshold: 0.1				
Culture	82.2% (71.5–90.2)	95.8% (89.0–99.6)	73/63	0.94
qPCR	89.5% (66.7–98.7)	25.4% (15.3–37.9)	19/63	0.60
Culture + qPCR	65.9% (55.3–75.6)	95.2% (86.7–99.0)	91/63	0.85
Culture + qPCR + MAT	58.7% (48.9–68.1)	95.2% (86.71–99.0)	109/63	0.79

AUC = area under the curve; CI = confidence interval; MAT = microscopic agglutination test; ROC = receiver operator characteristics; qPCR = quantitative real-time polymerase chain reaction.

* Number of positive/negative samples included in the ROC calculation.

† AUC quantifies the overall ability of the test to discriminate between those individuals with the disease and those without the disease (interpretation: 0.9–1 = excellent; 0.8–0.9 = good; 0.7–0.8 = fair; 0.6–0.7 = poor).^19^

**Table 2:** Sensitivity and specificity of BCF compared with blood when different inclusion criteria are applied for the BCF qPCR

		Venous blood qPCR		Final diagnostic characteristics for using BCF			
		Positive	Negative	Sensitivity (95% CI)	Specificity (95% CI)	PPV* (%)	NPV* (%)
	BCF: Cq ≤ 40						
BCF	Positive	3	4	30% (10.3–60.7)	99% (97–100)	43	98
	Negative	7	338				
	BCF: Cq ≤ 42						
	Positive	3	18	30% (10.3–60.7)	95% (92–97)	14	98
	Negative	7	324				
	BCF: Cq ≤ 45						
	Positive	5	41	50% (23.7–76.3)	88% (84–91)	11	98
	Negative	5	301				

BCF = blood culture fluid; CI = confidence interval; NPV = negative predictive value; PPV = positive predictive value; qPCR = quantitative real-time polymerase chain reaction.

* PPV is the proportion of positive results that are truly positive, and NPV is the proportion of negative results that are truly negative.
